# The therapeutic potential of RNA Polymerase I transcription inhibitor, CX-5461, in uterine leiomyosarcoma

**DOI:** 10.1007/s10637-022-01222-w

**Published:** 2022-02-24

**Authors:** Chang-Won Kang, Katherine M. Hannan, Anneke C. Blackburn, Amos H. P. Loh, Kuick Chik Hong, Goh Jian Yuan, Nadine Hein, Denis Drygin, Ross D. Hannan, Lucy A. Coupland

**Affiliations:** 1 ACRF Department of Cancer Biology and Therapeutics, The John Curtin School of Medical Research, The Australian National University, Canberra, ACT Australia; 2 VIVA-KKH Paediatric Brain and Solid Tumour Programme, KK Women’s and Children’s Hospital, Bukit Timah, Singapore; 3 Department of Pathology and Laboratory Medicine, KK Women’s and Children’s Hospital, Bukit Timah, Singapore; 4 Regulus Therapeutics, 4224 Campus Point C, San Diego, CA USA; 5 Department of Biochemistry and Molecular Biology, University of Melbourne, Parkville, 3010 Australia; 6 Department of Biochemistry and Molecular Biology, Monash University, Clayton, 3800 Australia; 7 School of Biomedical Sciences, University of Queensland, 4067, St Lucia, Australia

**Keywords:** Uterine leiomyosarcoma, SK-UT-1, RNA Polymerase I transcription inhibitor, CX-5461

## Abstract

*
Background.* Uterine leiomyosarcoma is a rare aggressive smooth muscle cancer with poor survival rates. RNA Polymerase I (Pol I) activity is elevated in many cancers supporting tumour growth and prior studies in uterine leiomyosarcoma revealed enlarged nucleoli and upregulated Pol I activity-related genes. This study aimed to investigate the anti-tumour potential of CX-5461, a Pol I transcription inhibitor currently being evaluated in clinical trials for several cancers, against the human uterine leiomyosarcoma cell line, SK-UT-1. *Methods.* SK-UT-1 was characterised using genome profiling and western blotting. The anti-tumour effects of CX-5461 were investigated using cell proliferation assays, expression analysis using qRT-PCR, and BrdU/PI based cell cycle analysis. *Results.* Genetic analysis of SK-UT-1 revealed mutations in *TP53*, *RB1*, *PTEN*, *APC* and *TSC1 & 2*, all potentially associated with increased Pol I activity. Protein expression analysis showed dysregulated p53, RB1 and c-Myc. CX-5461 treatment resulted in an anti-proliferation response, G2 phase cell-cycle arrest and on-target activity demonstrated by reduced ribosomal DNA transcription. *Conclusions.* SK-UT-1 was confirmed as a representative model of uterine leiomyosarcoma and CX-5461 has significant potential as a novel adjuvant for this rare cancer.

**Supplementary Information:**

The online version contains supplementary material available at 10.1007/s10637-022-01222-w.

## Introduction

Uterine leiomyosarcoma is a subtype of soft tissue sarcoma and arises in the smooth muscle of the uterus. Although rare, accounting for < 5% of female genital tract cancers, and the fact that 60% of cases are diagnosed at an early stage, it is an extremely aggressive and progressive cancer with a 5-year survival rate for localised disease of 52% and metastatic disease rate of 12% [1, 2]. Surgical resection and chemotherapy are used as the mainstay for uterine leiomyosarcoma management, however, the curative effects are limited [3–5]. In addition, loss of heterozygosity at loci containing the tumour suppressor genes, tumour protein 53 (*TP53)*, retinoblastoma 1 (*RB1*), phosphatase and tensin homolog (*PTEN*) and cyclin-dependent kinase inhibitor protein (*CDKN2A*), in uterine leiomyosarcoma confers chemotherapy resistance [6, 7]. There is an urgent need, therefore, to identify new targets and effective therapeutic strategies to improve the outcomes of this aggressive disease.

Rapid growth of cancers, one of the classic hallmarks, is associated with elevated rates of protein synthesis and ribosome biogenesis (RiBi) [8]. An early rate limiting step in RiBi, is the transcription of the 47S precursor ribosomal RNA (rRNA) by Pol I, which is rapidly processed into mature rRNA forming the nucleic backbone of ribosomes. RiBi occurs in the nucleolus at elevated levels in cancer cells to sustain the rapid growth rate [9, 10]. Enhanced Pol I transcription activity can be mediated by altered signalling pathways and genetic mutations in key oncogenes or tumour suppressors (PI3K/Akt, RAS/MAPK, mTOR, MYC, P53, RB1 and ATRX). It is for these reasons Pol I was considered a key target for cancer treatment and specific inhibitors were developed [11].

Genetic studies of uterine leiomyosarcoma tumours from patients report that genes related to Pol I transcription activity, including *TP53*, *RB1*, *ATRX* and *MYC,* are frequently mutated. *TP53* and *RB1* were confirmed as the most frequently altered genes with 61% and 48%, respectively, showing somatic mutations or homozygous deletions. *ATRX* was mutated in 34% and *MYC* was amplified in 38% of cases [7, 12]. Enlarged nucleoli, a marker of aggressive disease, and increased rDNA transcription have also been reported in uterine leiomyosarcoma [13]. Taken together, these studies suggest that inhibition of Pol I transcription may be an effective new therapeutic strategy for the treatment of uterine leiomyosarcoma. To investigate this hypothesis, (i) we analysed the genetic profile of the human uterine leiomyosarcoma cell line, SK-UT-1, to identify key driver mutations linked to Pol I activity and to establish the cell line as a representative in vitro model of this rare cancer type, hence developing a robust and suitable model system for testing novel therapies, and (ii), we evaluated the effects of the Pol I transcription inhibitor, CX-5461 [14–16], against this cell line.

## Methods

### Genomic profiling of SK-UT-1

The Ampliseq™ Cancer Childhood Panel DNA assay (Illumina) detects single nucleotide variants (SNV) from hotspots of 86 genes, full exons of 44 genes, and copy number variants (CNV) from 28 clinically relevant cancer genes. DNA was extracted from the cell lines using ReliaPrep tissue DNA extraction kit (Promega, Madison, WI, USA). The Childhood Cancer Research Assay primers and AmpliSeq Library Kit Plus (Illumina) were used for library preparation. The prepared libraries were sequenced using a MiniSeq sequencer (Illumina). Base calling and mapping to a reference genome (hg19) was performed using the BaseSpace Informatics suite (Illumina). SNV variant calling was performed in DNA amplicon application (Illumina, version 2.1.1). CNV calling was performed in OncoCNV caller application (Illumina, version 1.2.0). All VCF files were loaded into variant interpreter (version 2.7.0.412) for interpretation. SNV somatic candidates were selected based on minimum coverage reads of more than 100, minimum allele frequency of 10%, cosmic reported variant and less than 1% prevalence in the 1000 Genome Population Database. CNV candidates with more than 10 gene copies were identified.

### Cell culture and IncuCyte-based cell proliferation assay

Human uterine leiomyosarcoma cell line, SK-UT-1, was purchased from American Tissue Culture Collection (ATCC^®^, Manassas, VA, USA) and cultured with minimum essential medium (MEM) containing 10% Foetal Bovine Serum (FBS) (Sigma-Aldrich, St Louis, MO), 2 mM L-glutamine (Gibco, Waltham, MA), 1% Puromycin/Streptomycin/Neomycin (Gibco, Waltham, MA). 1 × 10^3^ of SK-UT-1 cells were seeded into a 96-well plate and incubated for 12 h (hr). The medium was then exchanged with fresh complete medium containing vehicle or various concentrations of CX-5461 and incubated for 72 h in a ZOOM IncuCyte^®^ Live Cell Imaging System. The confluence in each well was recorded every 12 h. The 50% growth inhibitory concentration (GIC_50_) of CX-5461 was determined by Graph-Pad Prism (Version 9.1.0). A human foreskin fibroblast cell line was purchased from Lonza (Lonza, Basel, Switzerland). The human foreskin fibroblast cells were cultured with Dulbecco’s modified eagle medium (DMEM) containing 10% of FBS, 8 mM L-glutamine, 1% Puromycin/Streptomycin/Neomycin.

### Western blotting

SK-UT-1 cells (1.0 × 10^6^) and human fibroblast cells (1.0 × 10^6^) were harvested, washed, then lysed in cell lysis buffer containing 0.5 mM EDTA, 2% SDS, 20 mM HEPES and protease inhibitor (Roche, Basel, Switzerland). The protein concentration was measured using a DC*™* protein assay kit (Bio-Rad, Hercules, CA) and 30 µg of protein was resolved by SDS-PAGE. Once separated, the proteins were transferred to a Polyvinylidene fluoride (PVDF) membrane (Bio-Rad, Hercules, CA) using the Bio-Rad semi-dry transfer protocol (1 mA current, 30 V constant voltage for 30 min). PVDF membranes were rinsed in 1X Tris-Buffer Saline Tween-20 (TBST) (1X TBS (pH7.5) 1 mM Tris, 10 mM NaCl, 0.1% Tween 20), then blocked with 5% non-fat dry milk in 1X TBST for 1 h at room temperature. The membrane was probed with primary antibodies (Supplementary Table 2), diluted in 5% BSA in 1X TBST, overnight at 4 °C then washed 1X TBST and incubated with horseradish peroxidase-conjugated (HRP) secondary antibodies (Supplementary Table 2) for 1 h at room temperature. The membranes were washed with 1X TBST, developed for enhanced chemiluminescence using Clarity Western ECL Substrate reagent (Bio-Rad, Hercules, CA), then the blots were imaged with a ChemiDoc MP Imager (Bio-Rad, Hercules, CA). β-actin was used as a loading control. Band intensity was analysed using Bio-Rad Image Lab Software (Version 6.0.1).

### Quantitative Real-Time PCR for measurement of rDNA transcription

rDNA transcription was determined by qPCR as previously described Bywater et al. [15]. Briefly, SK-UT-1 cells (1.0 × 10^5^) were seeded into 100 mm cell culture dishes and incubated for 12 h, following which the medium was exchanged with fresh complete medium containing vehicle or various concentrations of CX-5461, then incubated for 1 h. RNA was then extracted using a RNeasy^®^ Mini Kit (Qiagen, Hilden, Germany) according to the manufacturer’s instructions. RNA (500 ng) was DNase treated (according to kit instructions), then incubated in T100™ Thermocycler (Bio-Rad, CA, USA) using the settings detailed in Supplementary Table 3. cDNA synthesis was performed using SuperScript™ IV Reverse Transcriptase (Invitrogen, Carlsbad, CA), Hexamer Random Primers (Promega, Madison, WI), and dNTPs (Invitrogen, Carlsbad, CA) with incubation in a T100™ Thermocycler (detailed in Supplementary Table 3). RT-PCR was performed using SYBR™ Green master mix (Applied Biosystems, Foster City, CA) and primers (Supplementary Table 4) in a StepOne™ Plus Real Time (RT) PCR System (Applied Biosystems) with the settings detailed in Supplementary Table 3.

### BrdU and PI staining for cell cycle analysis

Bromodeoxyuridine (BrdU) and propidium iodide (PI) staining assay was performed following the manufacturer’s instructions. Briefly, 3 × 10^4^ cells were seeded in 100 mm cell culture dish and incubated for 12 h. The media was then changed to complete medium containing vehicle or CX-5461 at the GIC^50^ concentrations determined in the cell proliferation assay. After 72 h, 10 µM BrdU (Sigma-Aldrich, St Louis, MO) was added for 30 min and the cells harvested and stained with the anti BrdU specific antibody (Clone: B44, BD Bioscience, Franklin Lakes, NJ) and PI (Sigma Aldrich, St Louis, MO) according to the manufacturer’s instructions. Following the staining, samples were analysed using flow cytometry on a Becton Dickinson LSR II FACS machine and the data analysed using FlowJo software (Version 10.0).

### Statistical analysis

Statistical analyses were conducted using GraphPad Prism software (Version 9.1.0). Comparison of western blotting band intensities between SK-UT-1 and human fibroblast (HF) cell lines was conducted using a Mann–Whitney U test. Statistical significance between two groups was assessed by two-way ANOVA. Dose–response curve fit and 50% inhibitory values were determined using non-linear regression analysis.

## Results

### Pol I transcription-related genes are altered in the SK-UT-1 cell line

**Fig. 1:**
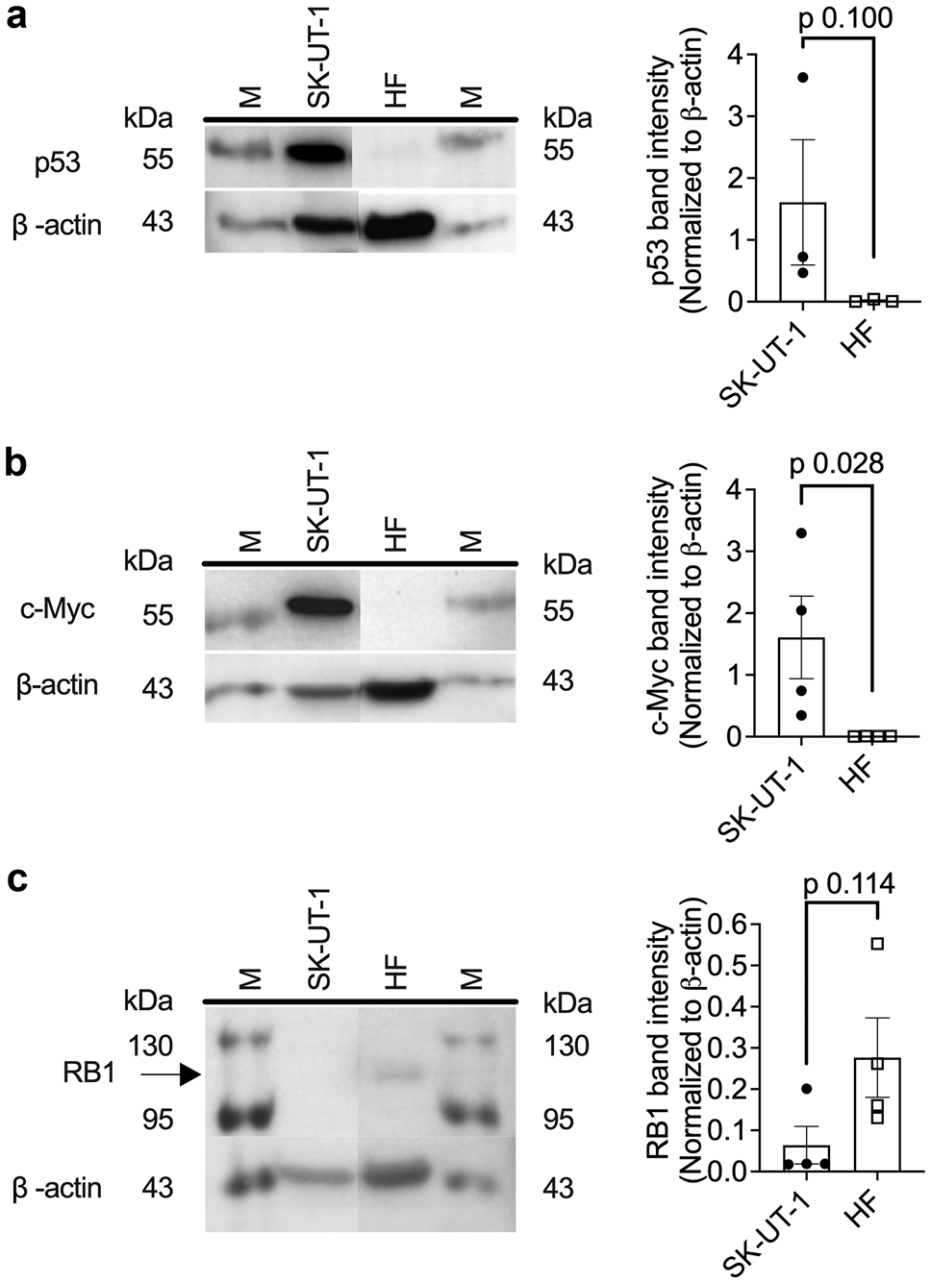
Basal expression of key proteins on uterine leiomyosarcoma SK-UT-1 cells. Basal protein abundance of (**a**) p53, (**b**) c-Myc and (**c**) RB1 were evaluated by western blot analysis. Human fibroblasts (HF) were used as a control cell line (note: intervening lanes with non-relevant cell lines have been removed from the image). M is abbreviated for marker lane. Band intensities were normalized to β-actin. Statistical analysis was conducted using a Mann Whitney U test (Error bars represent mean ± SEM of *n* = 3–4 biological replicates)

Genomic profiling of the SK-UT-1 cell line revealed missense, non-sense, frameshift and splice-site mutations in 21 genes (Supplementary Table 1). Consistent with prior uterine leiomyosarcoma genomic studies [7, 12], *TP53*, *RB1* and *PTEN* mutations were identified in the SK-UT-1 cells. Mutations were also found in Phosphatidylinositol 3-kinase (*PIK3CA*) and Adenomatous polyposis coli protein (*APC*) genes, that are reported to impact Pol I transcription activity [9, 10]. Deleterious mutations in the tumour suppressor genes, Tuberous Sclerosis Complex 1 (*TSC1*) and *TSC2*, were also identified which have not been previously reported to our knowledge. Western blotting analysis of protein lysate generated from SK-UT-1 cells demonstrated elevated levels of p53 and c-Myc protein, but undetectable levels of RB1 protein in comparison to the human fibroblast (HF) control cell line (Fig. 1a - c). These mutations were consistent with the expected sequelae of *TP53* gain of function hotspot mutations, R248Q and R175H, leading to stabilisation of p53, and *RB1* frameshift variant detected [17–19].

## CX-5461 inhibits uterine leiomyosarcoma cell proliferation

**Fig. 2:**
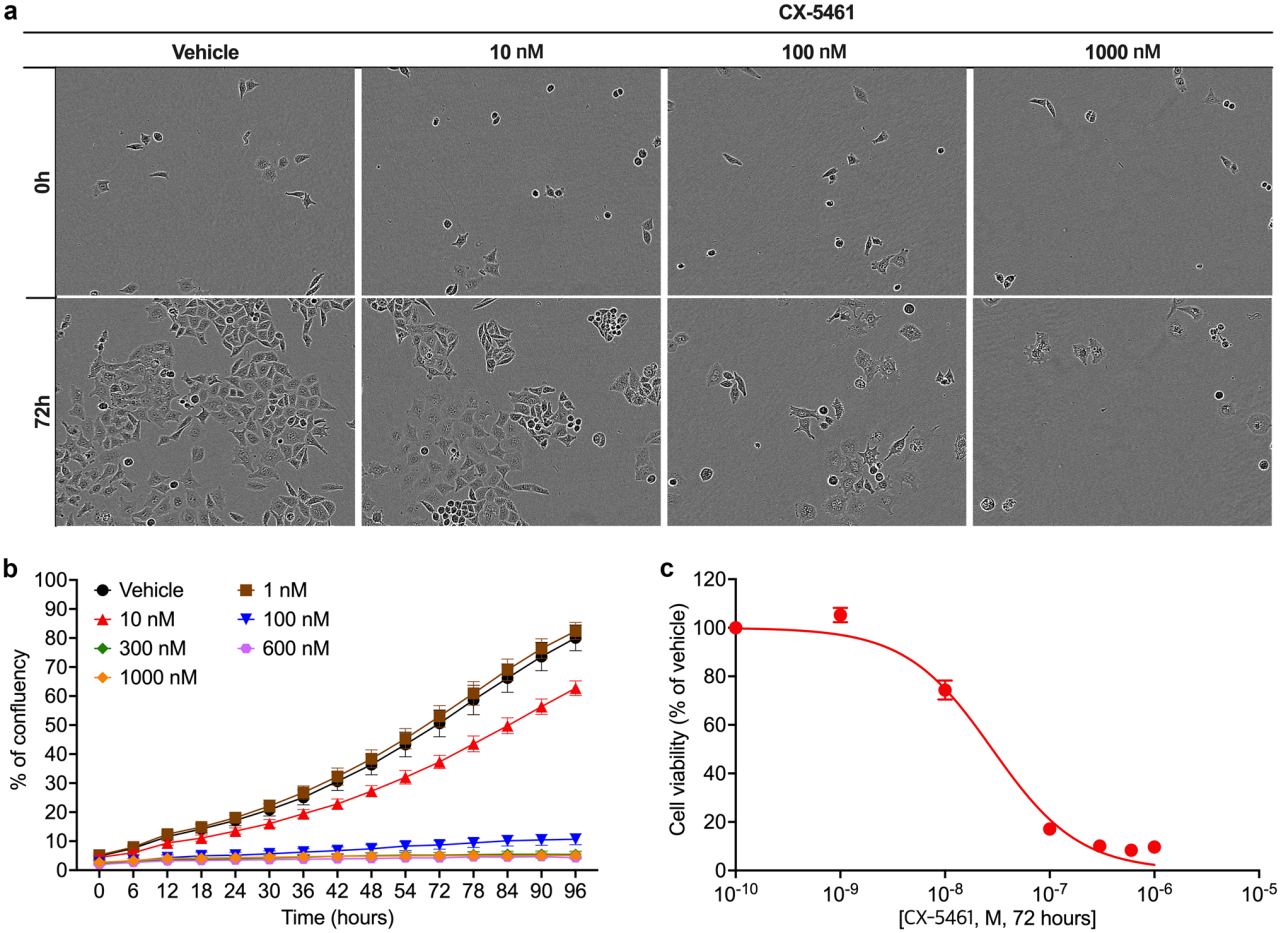
Anti-proliferation effects of CX-5461 on SK-UT-1 cells. (**a**) SK-UT-1 cells were treated with CX-5461, at the concentrations indicated, or vehicle (50 mM NaH_2_PO_4_ (pH4.5)) and assessed for proliferation via phase contrast microscopy and IncuCyte^®^ ZOOM Live Cell Imaging System (Error bars represent mean ± SEM of *n* = 3 biological triplicates). A dose–response curve (**c**), generated from data obtained in (**b**), with the 50% growth inhibitory concentration (GIC_50_) of CX-5461 calculated using non-linear regression analysis

The antitumour potential of Pol I transcription inhibitor CX-5461 on SK-UT-1 was assessed by a cell proliferation assay (Fig. 2). Treatment studies with CX-5461 demonstrated a dose-dependent decrease in cell density (% of confluency) following 72 h of drug exposure (Fig. 2a, b). The 50% growth inhibitory concentration (GIC_50_) was calculated at 28.1 ± 3.1 nM (Fig. 2c).

## rDNA transcription is targeted by CX-5461

**Fig. 3:**
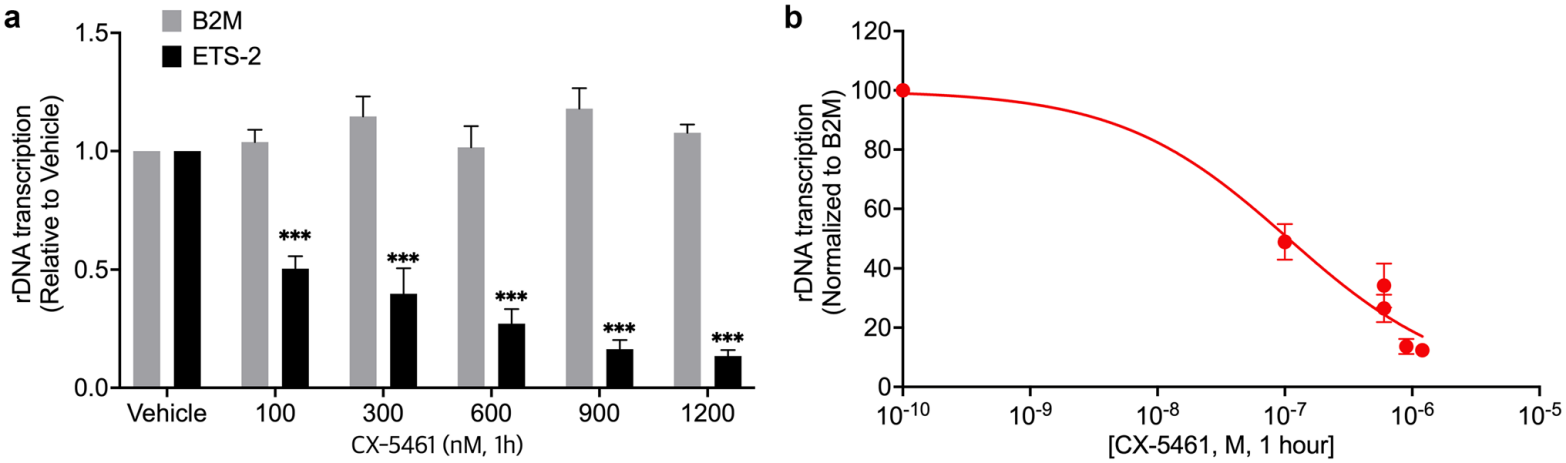
Inhibitory effects of CX-5461 on rDNA transcription in SK-UT-1 cells **(a)** The effects of treatment with CX-5461 at varying concentrations, and vehicle control, for 1 h on rDNA transcription rate in SK-UT-1 cells, determined via qPCR with the result of the external transcribed spacer (ETS) normalised to the house-keeping gene, β2 microglobulin (β2M). Statistically significance differences between ETS-2 and β2M were determined using two-way ANOVA (Error bars represent mean ± SEM of *n* = 3) *p <0.05, **p <0.01, ***p <0.001. (**b**) The 50% rDNA transcription inhibitory concentration (tIC_50_) of CX-5461 was calculated from (**a**) using non-linear regression analysis (Error bars represent mean ± SEM of *n* = 3 biological triplicates)

To investigate the effect of CX-5461 on its primary target, rDNA transcription, the abundance of 5’-External Transcribed Spacer (ETS), a rapidly processed region of the 47S pre-ribosomal RNA, was measured by q-RT-PCR after CX-5461 treatment (1 h). CX-5461 effectively inhibited rDNA transcription rate in a dose-dependent manner (Fig. 3a). The 50% transcription inhibitory concentration (tIC_50_) for CX-5461 was calculated at 112 ± 32 nM (Fig. 3b).

## CX-5461 induces G2 phase cell cycle arrest

**Fig. 4:**
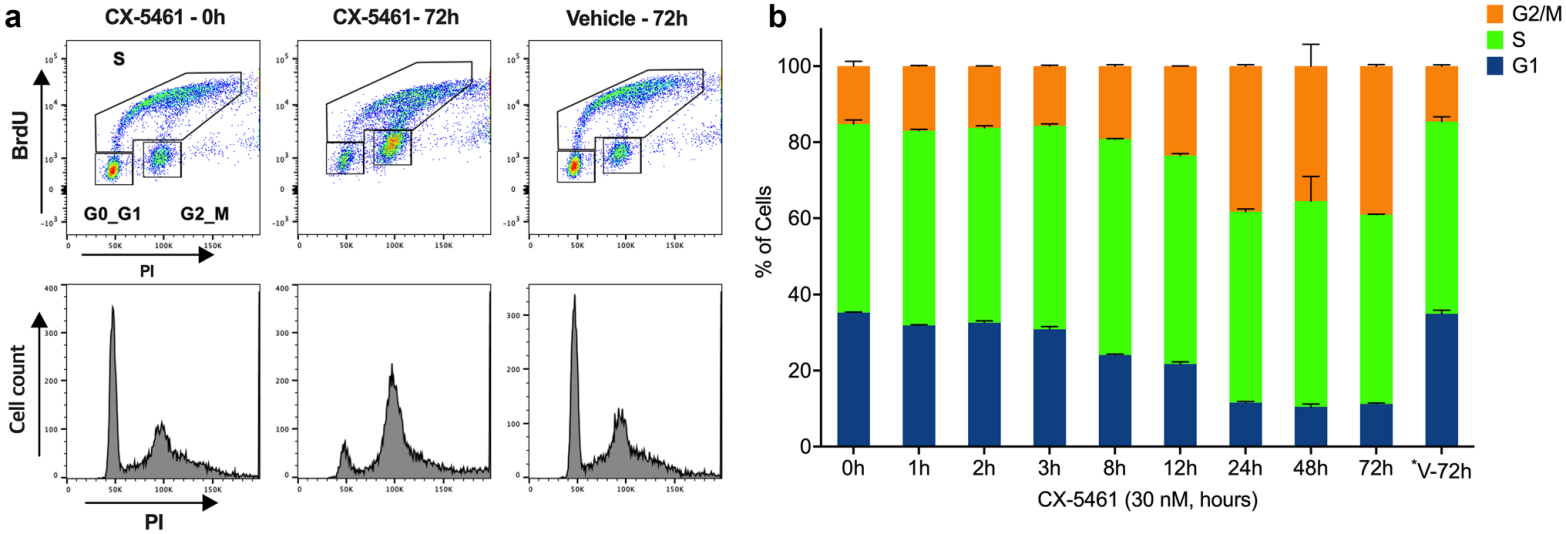
Effects of CX-5461 on cell cycle progression in SK-UT-1 cells (**a**) Flow cytometry gating strategy for cell cycle analysis using BrdU and PI staining at 0 and 72 h following treatment with 30 nM CX-5461, or vehicle, is shown. (**b**) SK-UT-1 cells were treated with the GIC_50_ CX-5461 dose (30 nM) for the times indicated, and stained with a anti BrdU specific antibody and PI for cell cycle analysis as in (**a**) (bars represent mean ± SD of *n* = 2 biological duplicates). *V-72 h: Vehicle for 72 h

SK-UT-1 was identified as p53 mutant in the genetic analysis and protein expression assays (Supplementary Table 1 and Fig. 1a). In the absence of wild-type p53, CX-5461 has previously been demonstrated to induce cell cycle defects that result in the accumulation of cells in the G2 phase of the cell cycle [20]. To investigate the effect of CX-5461 on SK-UT-1 cell cycle progression, BrdU and PI staining assays were conducted (Fig. 4a). Consistent with previous studies, cell cycle analysis revealed an increase in the percentage of cells in G2/M phase of the cell cycle and a reduction in the G1 subpopulation after 8 h of CX-5461 treatment (Fig. 4b). No change was detected in the percentage of cells in S phase at the treatment timepoints analysed.

## Discussion

This paper details the first studies evaluating the therapeutic potential of the Pol I transcription inhibitor, CX-5461, against uterine leiomyosarcoma. CX-5461, a first-in-class Pol I transcription inhibitor, selectively inhibits Pol I transcription by preventing pre-initiation complex formation at the rDNA promoter, and has demonstrated broad-spectrum anticancer therapeutic efficacy in multiple in vitro and in vivo studies of human cancers [21] (lymphoma [15], AML [22], neuroblastoma [23], breast cancer [24], osteosarcoma [25] and prostate cancer [26]). CX-5461 was well-tolerated in a recently completed phase I clinical trial in patients with haematological malignancy [16], and a phase II clinical trial for breast cancer (NCT02719977) is currently underway. Recently, Sanij et al.reported the therapeutic potential of CX-5461 in pre-clinical studies of ovarian cancer [27], however, the effects of CX-5461 on uterine leiomyosarcoma have not yet been evaluated.

Genes that influence Pol I transcription activity, such as *TP53*, *RB1*, *PTEN*, *ATRX and MYC,* have been found to be mutated in uterine leiomyosarcoma tumours, with *TP53* and *RB1* being the most-commonly altered genes [7, 12]. MYC regulates rDNA transcription through two mechanisms; firstly it directly transcriptionally upregulates the majority of the Pol I components [28]; secondly, it binds to rDNA promoter regions and promotes Pol I transcription through chromatin remodelling and interaction with Pol I-related cofactor [29]. In contrast, p53 and RB suppress Pol I transcription by disrupting Pol I binding to the rDNA gene promoter [30, 31].

Consistent with prior analysis [32, 33], our genomic profiling of the SK-UT-1 uterine leiomyosarcoma cell line confirmed mutations in *TP53*, *RB1, APC* and *PTEN*, but also the tumour suppressors *TSC1 and TSC2*. TSC1 and TSC2, together with the auxiliary subunit, Tre-Bud-Cdc16-1 domain member 7 (TBC1D), form the TSC complex [34] which regulates cell growth by controlling mTORC1 activation [35]. Loss of either *TSC1* or *TSC2* also increases c-Myc expression [35, 36], most likely as a direct result of mTORC1 signalling activation [37]. This would, in turn, enhance Pol I transcription activity [27, 38]. Evidence supporting a direct role for the TSC complex in this cancer type is that the allelic loss of *TSC2* in Eker mutant rats results in the spontaneous development of uterine leiomyosarcoma [39]. Western blotting studies in SK-UT-1 found elevated levels of p53 and c-Myc, and undetectable levels of RB1. In combination, these findings demonstrate that the SK-UT-1 cell line is broadly representative of uterine leiomyosarcoma tumours, and supports the likelihood that Pol I inhibition would be effective against this cancer type.

The major consequence of inhibiting rDNA transcription is activation of the nucleolar stress pathway through p53-dependent and p53-independent pathways [15, 20, 40]. CX-5461 has previously been shown to (i) activate a p53-dependent G1 phase arrest, in which cells may undergo apoptosis, cell cycle arrest, differentiation and/or senescence, and (ii) a p53-independent DNA damage response (DDR) [24, 27, 41]. Additionally, activation of the Ataxia telangiectasia mutated (ATM) and Ataxia telangiectasia and Rad3 related (ATR) kinase pathways by CX-5461 mediates a G2 phase cell cycle arrest [20]. In our studies, CX-5461 rapidly reduced the rate of rDNA transcription in p53 mutant SK-UT-1 cells following 1 h of treatment, with subsequent anti-proliferative effects observed from 12 h of treatment, presumably mediated via the observed G2 phase cell cycle arrest noted by 8 h of treatment. Relatively low nanomolar CX-5461 concentrations were required to achieve these effects with the GI_50_ and tIC_50_ values being similar to those reported for other sensitive cancer cell lines [15, 27]. Dose escalation and pharmacokinetic/dynamic studies of CX-5461 in humans with haematological malignancies revealed that, even at the lowest dose of 25 mg/m^2^, Pol I inhibition occurred in tumour cells [16]. The striking proliferation defect at low nanomolar concentrations in our vitro studies (tIC50 = 112 ± 32 nM) are within the same range as the concentrations obtained in vivo [16], thus suggesting that the drug concentrations used in this study are translatable to therapeutically effective in vivo concentrations.

In conclusion, these studies (i) confirm the SK-UT-1 cell line as a representative model of uterine leiomyosarcoma, and (ii) demonstrate that targeting dysregulated Pol I transcription in this cancer type with CX-5461 has significant potential as a novel adjunct therapy. To strengthen these findings, future studies could include additional uterine leiomyosarcoma cell lines and in vivo mouse models of uterine leiomyosarcoma. However, given the poor outcomes of this disease with current treatment, the targeted biological mechanism being common to many cancers, and the in-human experience already gained with CX-5461 against several other cancer types, small pilot trials of CX-5461 in combination with salvage chemotherapy could be justified in patients who have failed standard treatment regimes.

## Supplementary Information

Below is the link to the electronic supplementary material.

Supplementary file1 (DOCX 29 KB)

## Data Availability

Data will be made available upon request through the Corresponding Author.
