# Supplementary material for: The therapeutic potential of RNA Polymerase I transcription inhibitor, CX-5461, in uterine leiomyosarcoma
Source: Invest New Drugs. 2022 Feb 24;40(3):529–36. doi: 10.1007/s10637-022-01222-w (PMC9098598; doi:10.1007/s10637-022-01222-w)
Supplement: Supplementary file 1 — Supplementary file1 (DOCX 29 KB) [file 10637_2022_1222_MOESM1_ESM.docx]

**Supplementary Table 1. Genetic mutations in uterine leiomyosarcoma cell line, SK-UT-1**

| **Genes** | **Names** | **Mutation status** | **Consequence** |
| --- | --- | --- | --- |
| ***Tumour suppressors*** | | | |
| TP53 | Tumour protein p53 | p.Arg248Gln | Missense_variant |
|  |  | p.Arg175His | Missense_variant |
| APC | Adenomatous polyposis coli protein | p.Gln1096Ter | Stop_gained |
|  |  | p.Thr1556LeufsTer9 | Frameshift_variant |
| RB1 | Retinoblastoma 1 | p.Val654CysfsTer4 | Frameshift_variant |
| ARID1A | AT-rich interactive domain-containing protein 1A | p.Gly276GlufsTer87 | Frameshift_variant |
|  |  | p.Pro1326ArgfsTer155 | Frameshift_variant |
| ARID1B | AT-rich interaction domain 1B | p.Gln1899Ter | Stop_gained |
|  |  | p.Gln2074Ter | Stop_gained |
| PTEN | Phosphatase and tensin homolog | p.Asn323LysfsTer2 | Frameshift_variant |
|  |  | p.Thr319Ter | Frameshift_variant |
| NSD2 | Nuclear Receptor Binding SET Domain Protein 2 | p.Pro1343GlnfsTer49 | Frameshift_variant |
| TSC1 | Tuberous sclerosis-1 | c.2626-7_2626-4delTTTT | Splice_region_varitant, intron_variant |
|  |  | p.Pro613Ser | Missense_variant |
| TSC2 | Tuberous sclerosis-2 | p.Gln90Ter | Stop_gained |
|  |  | p.Ala1195Val | Missense_variant |
| GATA1 | GATA -binding protein 1 | p.Arg113Cys | Missense_variant |
| GATA3 | GATA-binding protein 3 | p.Tyr283Cys | Missense_variant |
| KMT2D | Histone-lysine N-methyltransferase 2D | c.10441-11_10441-8delTCTC | Splice_region_variant, intron_variant |
|  |  | p.Arg1601His | Missense_variant |
|  |  | p.Pro647HisfsTer283 | Frameshift_variant |
| DICER1 | ribonuclease III | c.1510-4delt | Splice_region_variant, intron_variant |
| SUZ12 | SUZ12 polycomb repressive complex 2 subunit | c.823+15delT | Splice_region_variant, intron_variant |
| GNA13 | G protein subunit alpha 13 | p.Ser239Asn | Missense_variant |
| ***Oncogenes*** | | |  |
| PI3KCA | Phosphatidylinositol 3-kinase | p.Arg88Gln | Missense_variant |
| ALK | Anaplastic lymphoma kinase | p.Arg1192Gln | Missense_variant |
| EGFR | Epidermal Growth Factor Receptor | p.Ala653ProfsTer52 | Frameshift_variant |
| RHOA | Ras homolog gene family member A | p.Arg5Gln | Missense_variant |
| HRAS | HRas proto-oncogene | p.Arg73Cys | Missense_variant |

**Supplementary Table 2.** Antibodies used in western blotting

| **Primary Antibodies** | | | |
| --- | --- | --- | --- |
| **Human Target Protein** | **Raised in** | **Company** | **Catalogue number** |
| P53 | Mouse | Santa Cruz, Dallas, TX | SC-126 |
| RB1 | Rabbit | Santa Cruz, Dallas, TX | SC-0 |
| c-Myc | Mouse | Calbiochem, San Dieago, CA | OP10L |
| β-actin | Rabbit | Cell Signaling Technology, Danvers, MA | 4970S |
| **Secondary Antibodies** | | | |
| **Target Protein** | **Raised in** | **Company** | **Catalogue number** |
| Rabbit IgG  (HRP conjugated) | Goat | Bio-Rad, Hercules, CA | 1706515 |
| Mouse IgG  (HRP conjugated) | Goat | Bio-Rad, Hercules, CA | 1706516 |

HRP: horseradish peroxidase

mAb: monoclonal antibody; IgG=Immunoglobulin G

**Supplementary Table 3**. Incubation settings for Quantification Real-Time PCR.

|  | **Time and Temperature setting** |
| --- | --- |
| **DNase treatment** | 37° C for 15 min -> 70° C for 15 min -> 4° C |
| **cDNA synthesis** | 20° C for 5 min -> 37° C for 5 min -> 42° C for 75 min -> 70° C for 15 min -> 4° C -> Store at -20° C |
| **RT-PCR** | Holding stage: 95° C for 20 sec  Cycling Stage (40 cycles): 95° C for 3 sec -> 60° C for 30 sec  Melt curve stage (Melt curve 0.7): 95° C for 15 sec -> 60° C for 1 min -> 95° C for 15 sec |

**Supplementary Table 4**. Primer Sequences used in qPCR.

| **Name of primer** | **Sequence** |
| --- | --- |
| ETS-2 forward | 5’-GGCGGTTTGAGTGAGACGAGA-3’ |
| ETS-2 reverse | 5’-ACGTGCGCTCACCGAGAGCAG-3’ |
| β2M forward | 5’-CCGTGGVVTTAGCTGTGCTCGC-3’ |
| β2M reverse | 5’-CCCACTTAACTATCTTGGGCTG-3’ |
